# Hematogenous extraneural metastasis of the germinomatous component of a pineal mixed germ cell tumor

**DOI:** 10.1007/s10014-011-0080-y

**Published:** 2012-01-28

**Authors:** Megumi Asanuma, Tatsuro Aoyama, Keiichi Sakai, Koji Asano, Tsuyoshi Uehara, Kazuhiro Hongo

**Affiliations:** 1 Department of Neurosurgery, Shinshu University School of Medicine, Asahi 3-1-1, Matsumoto, 390-8621 Japan; 2 Department of Neurosurgery, National Hospital Organization Shinshu Ueda Medical Center, Midorigaoka 1-27-21, Ueda, 386-8610 Japan; 3 Department of Laboratory Medicine, Shinshu University Hospital, Asahi 3-1-1, Matsumoto, 390-8621 Japan

**Keywords:** Pineal germ cell tumor, Hematogenous metastasis, Autopsy

## Abstract

A 23-year-old man presented with a mass in the pineal region and obstructive hydrocephalus. A neuroendoscopic biopsy for the lesion, ventriculoperitoneal (VP) shunting, and focal irradiation were conducted as initial treatment. Histological diagnosis of the biopsy specimen was germinoma. He underwent further irradiation and two tumor resections. Histological diagnosis was mature teratoma without a germinomatous component. After serial treatments, the intracranial lesion was controlled. However, 14 months after presentation, extraneural lesions were confirmed in the posterior mediastinum and retroperitoneal space. The biopsy specimen of the retroperitoneal space lesion was histologically diagnosed as germinoma. Although chemotherapy with cisplatin and etoposide was undertaken, extraneural lesions ware uncontrollable and he died. At autopsy, extraneural lesions were confirmed in the posterior mediastinum, retroperitoneal space, and right lung. Histological diagnosis of extraneural lesions was germinoma. This case was considered to be a pineal mixed germ cell tumor mainly consisting of germinoma and mature teratoma, which caused hematogenous metastasis of the germinoma component. Systemic chemotherapy and irradiation for primary lesions as an initial treatment is important to cure the primary lesion and prevent extraneural metastasis.

## Introduction

Extraneural metastases of primary brain tumors are rarely reported. Extraneural metastasis of primary brain tumor is thought to occur in situations such as direct local invasion, hematogeneous or lymphatic spread, and seeding via the cerebrospinal fluid pathways. The most common such brain tumor is glioblastoma, followed by medulloblastoma and ependymoma. There are few reports of patients with hematogeneous metastasis associated with intracranial germ cell tumors [1–3]. Most of the cases in the literature are malignant nongerminomatous tumors. Hematogenous metastasis from germinoma is rare. We report a case of a pineal mixed germ cell tumor mainly consisting of germinoma and mature teratoma, confirmed by autopsy, with hematogenous metastasis of the germinoma component.

## Case report

### Clinical summary

**Fig. 1:**
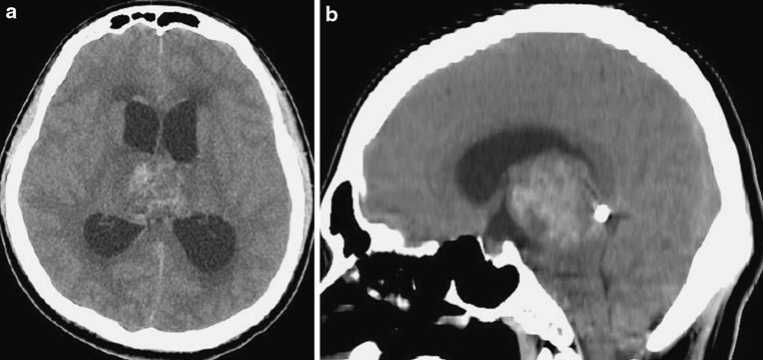
Initial plain CT scans (**a** axial image, **b** sagittal image) showing a mixed density mass with calcification in the pineal region and hydrocephalus

**Fig. 2:**
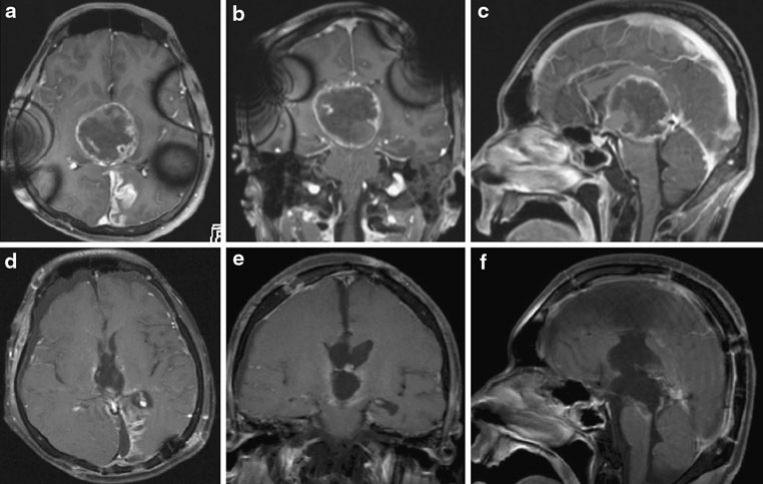
MR images taken during the course. **a**–**c** MRI with contrast during radiation showing enlargement of the tumor, indicating growing teratoma syndrome. **d**–**f** MRI with contrast after the third surgery showing a residual lesion on the right side of the third ventricle

A 23-year-old man was transferred to our department because of progressive consciousness disturbance. He was drowsy on admission. A CT scan revealed a well-demarcated and heterogenous mass with calcification in the pineal region. The mass was approximately 40 mm in diameter, and extended into the lateral ventricles, causing obstructive hydrocephalus (Fig. 1). Based on the CT scan, a non-germinomatous germ cell tumor was suspected. The patient developed Cushing’s sign soon after admission. A neuroendoscopic biopsy was conducted for the lesion and ventriculoperitoneal (VP) shunting was performed. Endoscopic third ventriculostomy was not selected because the third ventricle was filled with the tumor. The lesion was reddish in color; histological diagnosis of the biopsy specimen was germinoma. Serum AFP concentration was 7.4 ng/ml (normal <10 mg/ml). HCG level was 4.6 mIU/ml (normal <1.0 mIU/ml), and HCG-β level was ≤0.1 ng/ml. In the cytological examination of cerebrospinal fluid, tumor cells were not detected. His consciousness disturbance persisted after the surgery. Although he subsequently underwent focal irradiation (20 Gy), the tumor enlarged. Two weeks after presentation, during irradiation, he developed deep venous thrombosis and pulmonary embolism. A filter was placed in the inferior vena cava and anticoagulant was administered. Systemic CT scan did not show abnormal mass lesions except for venous thrombosis. Three weeks after presentation, partial resection was carried out via an occipital interhemispheric transtentorial approach. Histological diagnosis was mature teratoma. The germinomatous component was not identified. Tumor enlargement during radiation was considered to be “the growing teratoma syndrome”. Whole-brain irradiation (24 Gy) was administered postoperatively. After tumor resection and irradiation, serum AFP concentration was to 2.4 ng/ml and HCG level was <1.0 mIU/ml. Three months after presentation, subtotal removal was carried out via an anterior transcallosal transchoroidal approach. The shunt system was also removed, because obstructive hydrocephalus improved. Histological diagnosis was mature teratoma without a germinomatous component. Serum AFP concentration was 1.5 ng/ml and HCG level was <1.0 mIU/ml after subtotal resection. In a series of cerebrospinal fluid examinations, tumor cells were not detected. The residual lesion was controlled (Fig. 2). His consciousness disturbance failed to improve, and he was bedridden.

**Fig. 3:**
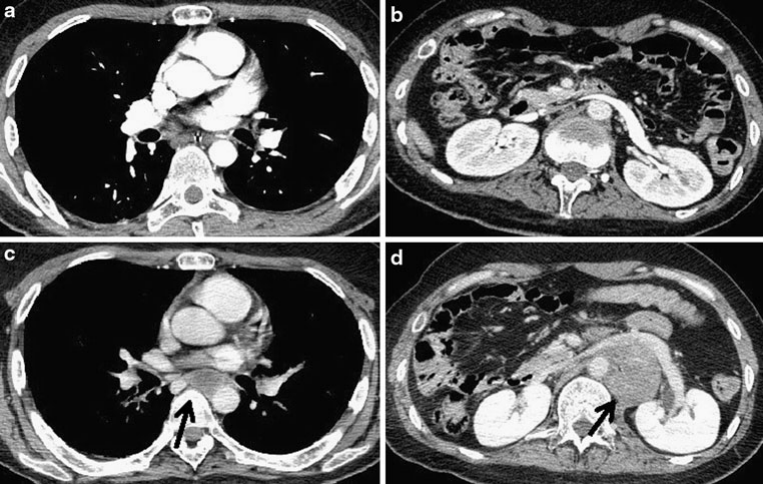
Chest and abdominal CT scans with contrast. **a**, **b** Nine months after presentation showing no lesions. **c**, **d** Fourteen months after presentation showing tumors in the posterior mediastinum and retroperitoneal space (*arrows*)

Fourteen months after presentation, chest and abdominal CT scans for examination of unknown fever revealed masses in the posterior mediastinum and retroperitoneal space (Fig. 3). Serum AFP concentration was 2.2 ng/ml and HCG level increased to 40.9 mIU/ml. CT-guided biopsy was conducted for the retroperitoneal space lesion; histological diagnosis was germinoma. The lesions enlarged over the course of a month. Systemic chemotherapy with cisplatin 10 mg/m^2^ and etoposide 50 mg/m^2^ was undertaken, but discontinued because of allergy and poor general condition. Although the lesions transiently reduced in size during treatment, they enlarged rapidly when treatment was discontinued, coinciding with a sudden deterioration of the patient’s general condition. Eighteen months after presentation, the patient died due to sudden shock. At autopsy, the cause of death was stated as hemorrhagic shock due to an intratumoral hemorrhage in the retroperitoneal space lesion.

### Histopathological findings

**Fig. 4:**
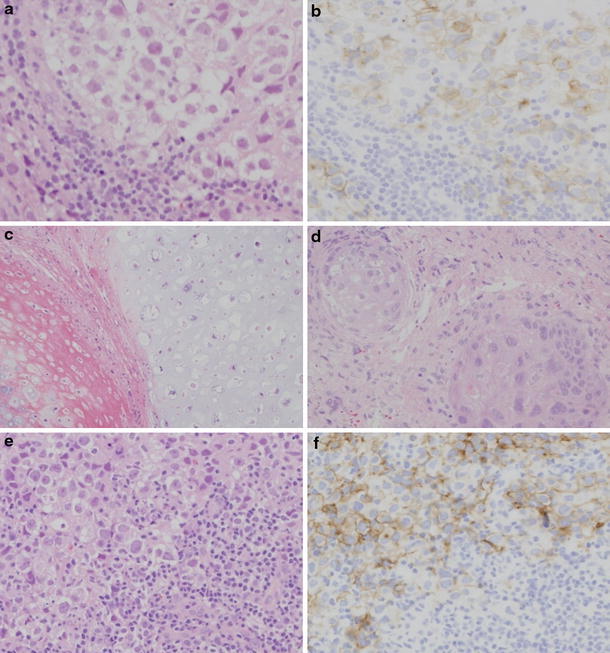
Photomicrographs of histological findings. **a**, **b** Initial biopsy specimen showing two-cell pattern of germinoma with large clear cells and small lymphoid elements. Immunohistochemical staining for PLAP revealed positive cells. (**a** H&E stain, **b** PLAP ×100). The specimens from the second surgery showed mature teratoma features with cartilage (**c**) and stratified squamous epithelium (**d**) (H&E stain ×200). **e**, **f** Biopsy specimen from retroperitoneal space lesion showing two-cell pattern of germinoma. Immunohistochemical staining for PLAP revealed positive tumor cells (**e** H&E stain, **f** PLAP ×200)

The biopsy specimen of the intracranial lesion showed the characteristic two-cell pattern of a germinoma with large clear cells and small lymphoid elements (Fig. 4a). Immunohistochemical staining for placental alkaline phosphatase (PLAP) (Fig. 4b) and c-kit revealed positive cells. The MIB-1 labeling index was 10–15%. The specimens from the second and third surgery showed mature teratoma features with cartilage, stratified squamous epithelium and glandular elements (Fig. 4c, d). The germinomatous component was not identified. The biopsy specimen from the metastatic abdominal lesion showed the stereotyped two-cell pattern of the germinoma, and immunohistochemical staining for PLAP revealed positive cells (Fig. 4e, f).

**Fig. 5:**
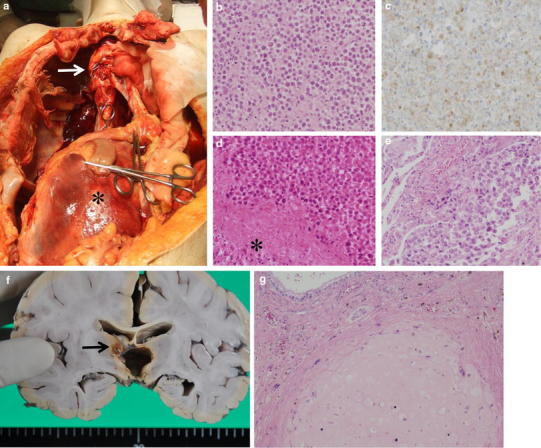
Macro and micrographs at autopsy. **a** Metastatic lesions of the posterior mediastinum (*arrow*) and retroperitoneal space (*asterisk*). Heart, lungs, liver, stomach, small intestine and large intestine were resected. The retroperitoneal space lesion (*asterisk*) encased the aorta, left adrenal grand, and the duodenum. **b**, **c** Histological findings from the posterior mediastinal lesion showing large tumor cells of germinoma. Immunohistochemical staining for PLAP revealed positive cells (**b** H&E stain, **c** PLAP ×200). **d** Histological findings of retroperitoneal space lesion showing degenerated tumor cells and adrenal tissue (*asterisk*). **e** Histological findings of lung lesion. **f** Residual lesion on the right side of third ventricle (*arrow*). **g** Histological findings of residual tumor showing mature teratoma features with cartilage and glandular elements (H&E stain ×200)

At autopsy, macroscopic observation showed that extraneural lesions were found in the posterior mediastinum, retroperitoneal space, and right lung (Fig. 5a).

The posterior mediastinal lesion was 8 cm × 6 cm in size, and located posterior to the tracheal bifurcation. The mass contained whitish necrotic tissue. The retroperitoneal space lesion was 17 cm × 15 cm in size and was associated with intratumoral hemorrhage. The mass encased retroperitoneal structures such as the aorta, left adrenal gland, and duodenum. The pulmonary lesion was in the right middle lobe. Histologically, extraneural lesions were diagnosed as germinoma (Fig. 5b–e). There were no morphological abnormalities or tumor cells in other organs, including the gonads. As a result, the patient was diagnosed as having a mixed pineal germ cell tumor, with extraneural metastasis of the germinomatous component to the posterior mediastinum, retroperitoneal space, and lung. The residual tumor of the brain was found on the right lateral wall of the third ventricle (Fig. 5f). There were no other lesions in the brain. Histological examination of the residual lesion revealed mature teratoma without a germinomatous component (Fig. 5g).

## Discussion

Germ cell tumors occasionally disseminate to the ventricular system and spinal subarachnoid spaces [4]. However, metastasis outside the central nervous system is rare. The incidence of metastasis via shunt tube is 10% [2]. Hematogenous metastasis occurs in only 3–5% of cases, and 7 cases of spinal epidural metastasis have been reported [1–3]. Most cases are non-germinomatous germ cell tumors. Histologically, choriocarcinomas are frequent [5].

Sugiyama et al. [6] investigated the relationship between tumor histology and serum AFP and HCG levels. They reported that in patients with serum AFP or HCG < 9.9 the main histological component was germinoma, 10.0 < AFP or HCG < 999 patients had teratomas, and 1000 < AFP or HCG patients had highly malignant yolk sac tumors or choriocacinomas. Serum AFP or HCG in our patient belonged to the AFP or HCG < 9.9 group; however, we could not establish a histologial diagnosis because we only performed a biopsy before irradiation. Therefore, the diagnosis of our case was considered to be a mixed germ cell tumor mainly consisting of germinoma and mature teratoma.

Although our patient underwent VP shunting, metastasis via shunt tube is deniable because the metastatic lesions arose in the retroperitoneal space, and there were no lesions in the abdominal cavity at autopsy. In some patients with primary intracranial germ cell tumors, metachronous lesions in the midline structures have been reported. According to the literature, metachronous lesions developed in the different site of the brain, mediastinum, or testis 8 months to 16 years after complete remission of the primary intracranial lesion [7–9]. As far as we are aware, metachronous multiple occurrence of germ cell tumors has not been reported. Remission of the primary pineal lesion could not be achieved for our case, and the retroperitoneal space and posterior mediastinal lesions were found 11 months after last surgery. The time interval between the initial and second lesions was short compared with previous metachronous cases. Ultimately, the lesion was also confirmed in the lung. Therefore, in our case, extraneural lesions were thought to be hematogenous, rather than metachronous, metastasis. Multiple surgery, including endoscopic biopsy, VP shunting, and craniotomies were regarded as possible factors in the hematogeneous spread.

Hematogenous metastasis from germinoma is rare, only 13 cases have been reported [1, 10–21]. These were ten male patients and three females, aged 9 months to 52 years (median 17.5 years). The duration to metastasis from the initial treatment ranged from 0 to 10.5 years (median 2.4 years). The most common metastatic site was bone, followed by lung, lymph nodes, and soft tissue. Although radiation was used as initial treatment for 11 of the 13 patients, chemotherapy was not performed in all patients. Most patients underwent chemotherapy after metastasis was confirmed. Eight of 13 patients were dead at a time of reporting. The median time to death from metastasis was 4.5 months (range 1 month to 1 year). Hematogenous metastasis from germinoma resulted in a poor outcome. The mechanism of metastasis remains unclear. Previous reports proposed that surgical damage to the blood–brain barrier and immunosuppression by repeated irradiation or chemotherapy may have contributed to the events leading to metastasis [2].

Germinomas are often cured with radiation. In our case, the germinomatous component in the primary region disappeared after radiation. However, metastasis occurred 14 months after presentation. Chemotherapy could not be used as the initial treatment because the patient’s condition was poor. Chemotherapy with cisplatin and etoposide were ineffective because of an adverse allergic reaction and poor general condition, but the lesions transiently diminished in size during the chemotherapy. According to previous reports, chemotherapy was effective for patients with germ cell tumor with metastatic extraneural lesions [1, 14]. However, metastatic lesions are difficult to cure and tend to be associated with poor outcome. Recently, the standard protocol for treatment of germ cell tumors has been chemotherapy followed by radiotherapy [22, 23]. Chemotherapy has not been used for all patients with hematogenous metastasis from germinoma, including our case. Germinomas have often been cured with radiation alone. Chemotherapy and irradiation as initial treatment for primary lesions is important to cure the primary lesion and prevent extraneural metastasis.
